# Microenvironments Matter: Advances in Brain-on-Chip

**DOI:** 10.3390/bios13050551

**Published:** 2023-05-16

**Authors:** Gulden Akcay, Regina Luttge

**Affiliations:** 1 Neuro-Nanoscale Engineering, Department of Mechanical Engineering/Microsystems, Institute of Complex Molecular Systems, Eindhoven University of Technology, 5600 MB Eindhoven, The Netherlands; g.akcay@tue.nl; 2 Eindhoven Artificial Intelligence Systems Institute, Eindhoven University of Technology, 5600 MB Eindhoven, The Netherlands; 3 Eindhoven Hendrik Casimir Institute, Eindhoven University of Technology, 5600 MB Eindhoven, The Netherlands

**Keywords:** Brain-on-Chip, instructive microenvironment, microfabrication

## Abstract

To highlight the particular needs with respect to modeling the unique and complex organization of the human brain structure, we reviewed the state-of-the-art in devising brain models with engineered instructive microenvironments. To acquire a better perspective on the brain’s working mechanisms, we first summarize the importance of regional stiffness gradients in brain tissue, varying per layer and the cellular diversities of the layers. Through this, one can acquire an understanding of the essential parameters in emulating the brain in vitro. In addition to the brain’s organizational architecture, we addressed also how the mechanical properties have an impact on neuronal cell responses. In this respect, advanced in vitro platforms emerged and profoundly changed the methods of brain modeling efforts from the past, mainly focusing on animal or cell line research. The main challenges in imitating features of the brain in a dish are with regard to composition and functionality. In neurobiological research, there are now methods that aim to cope with such challenges by the self-assembly of human-derived pluripotent stem cells (hPSCs), i.e., brainoids. Alternatively, these brainoids can be used stand-alone or in conjunction with Brain-on-Chip (BoC) platform technology, 3D-printed gels, and other types of engineered guidance features. Currently, advanced in vitro methods have made a giant leap forward regarding cost-effectiveness, ease-of-use, and availability. We bring these recent developments together into one review. We believe our conclusions will give a novel perspective towards advancing instructive microenvironments for BoCs and the understanding of the brain’s cellular functions either in modeling healthy or diseased states of the brain.

## 1. Introduction

The brain intrigues scientists in many aspects, with the study of consciousness as probably one of the most curiosity-driven of all brain research topics [1]. More practically orientated, it has become possible to reconstruct essential brain functions from human pluripotent stem cells (hPSCs) using either undirected or directed differentiation protocols [2,3,4]. Brain tissues resulting from the application of such culture protocols are often called minibrains, brainoids, or brain organoids, resembling structural features and cell distributions with specificity for particular brain sub-regions aiming for neurons reminiscent for their functional behavior of brain cells in vivo [5,6]. These types of cultures could present us with remarkable progress in understanding brain functions and mechanisms of neurodegenerative diseases, enabling new therapeutic interventions. However, the human brain’s unique complexity brings considerable challenges for creating an engineered brain model based on hPSC technology. At the current state-of-the-art, these exciting new brain models suffer from insufficient maturity to be useful in the study of an adult’s brain functions and its diseases [7]. Among others, the challenges coming with the needs for a medically relevant model have been addressed in a previous review by Bang et al. [8] and also recently in Maoz’ Perspective article on Brain-on-Chips (BoCs) [9].

In line with this reasoning, the understanding of human brain functions and recapitulating in vivo conditions of adult diseases requires novel and robustly engineered in vitro platforms. Efforts in improving technical platforms and chip-based assays should be guided by the merged activities in tissue engineering and microfluidics that already took place in developing Organ-on-Chip (OoC) models over the last 25 years [10]. Hence, one such technological solution is the design of a BoC utilizing microfabrication methods such as photolithography and thin-film technologies adapted from the semiconductor industry in replica molding techniques [11], which may include complementary microsystems technologies, such as laser cutting, ablation, micromilling, or 3D printing. Specifically, out-of-cleanroom microsystem technologies are pivotal when a quick mold fabrication scheme is needed for the replication of design variants of microfluidic chips from polydimethylsiloxane (PDMS) as one of the most common chip materials in OoCs. Biological experiments can require many more than nine repetitions in culture (at least three samples in the same culture run to sustain technical replicas from the same unfrozen passage number of the cell source and three independent runs per passage number) for a high enough statistical significance during the prototyping of designs, although this is a requirement often not yet aimed for in academia.

Cost-effective replication techniques or direct-write 3D printing are favored techniques for a quick turnaround in OoC design studies. As just mentioned, 3D printing is a rapid prototyping technique that came into the picture not only for the fabrication of molds but also for its direct exploitation in culture-ware as well as a means of biofabrication using cell laden gels. From the Maoz group, Rauti et al., for example, demonstrated this trend for 3D printed microfluidics in an elegant, rapidly prototyped modular concept of transforming a well into a chip, including cultures of brain cells [12].

Engineering of relevant brain models is requested by many clinical researchers since the majority of their research is performed in animal models that are known not to be the best models when it comes to genetically routed human diseases of the brain [13]. Throughout medical history, undoubtedly, animal models made crucial contributions to comprehend human diseases and still do, for example, in vaccine development [14]. More recently, it is becoming evident that conclusions gathered from animal research cannot be simply translated to human research [13,15].

In this respect, there is an urgent unmet need for technically and economically viable and relevant in vitro platforms for brain modeling. One way forward to overcome the above-described challenges is that engineered Brain-on-Chips could imitate structural features of the brain, regarding cell composition and the functionality of cell–cell interactions within a mimicry of the extracellular matrix (ECM). In neurobiological research, methods that encompass human-derived, induced pluripotent stem cells (hiPSCs) [16,17,18], brainoids [19,20,21], brain models based on 3D printed gels [22], brain interfaces [23], and sophisticated microfluidic BoC platforms [24] are on the verge of becoming attractive model systems. Thus far, the technologies being developed in this field had limited possibilities and remained a spearhead topic in academia and research institutes, but a remarkable increase in industrial interest by start-ups in this field [25,26,27] has enhanced their accessibility.

After this general introduction into the research field of brain models, the purpose of this review is to emphasize the special need for the engineering of instructive microenvironments in BoCs by taking into consideration the naturally occurring structural organization of the human brain. In Section 2, we first present such an understanding of the brain’s complex tissue microenvironment also by reviewing knowledge from animal models and early ex vivo characterization of brain slices addressing the brain’s layers, cellular diversity, and regional stiffness of its tissue. Subsequently, in Section 3, we discuss how the brain’s mechanical properties have an impact on neuronal cell behavior, and, in Section 4, microfluidic BoCs endowed with such an instructive microenvironment, thanks to applying advances in microfabrication, are introduced from the last 5 years of Brain-on-Chip research. Hence, Section 4 compiles an overview of selected BoC examples, which are representative for the four main technical developments in BoCs: (i) Microfluidic perfusion to mimic vascular flow, (ii) integrated perfused vascular tissue to mimic the blood–brain barrier, (iii) integrated 3D engineered scaffolds enabling organization and mimicry of layers of the brain, as well as (iv) compartmentalization and integrated organoids. Finally, we will conclude our findings from the literature in Section 5.

## 2. Brain Architecture: An Overview of the Naturally Appearing Microenvironment

The human brain is a complex organized system made up of billions of neurons and glial cells that create an elaborate and committed design of circuitry in every individual. This complexity comes into play throughout embryonic development, in which cells are self-organized into hierarchically organized structures [28,29]. At this neural development stage, cells begin to move into place forming a spatial distribution called a neural tube. The neural tube has highly populated neuroepithelial cells known as neural stem cells [30]. Neural stem cells are able to renew themselves while having the capability to form neurons, astrocytes, and oligodendrocytes in the process of neurogenesis [31]. Right after neurogenesis, neuronal migration, and differentiation by means of the cells’ neurite processes occur, consequently, the formation of dendrites and axons is followed-up by synaptogenesis [32]. As the new pluripotent cells are produced, they migrate to various brain regions, where they make new connections to establish neural networks upon differentiation triggered by the local microenvironment [33]. All processes including differentiation of the neural stem cells into multitude of different precursor cell types and their migration into the appropriate position is crucial to the development of healthy brains [34]. In more detail, the layering and stiffness gradients guide such processes, as depicted in the following sub-sections.

### 2.1. Brain Layers

**Figure 1:**
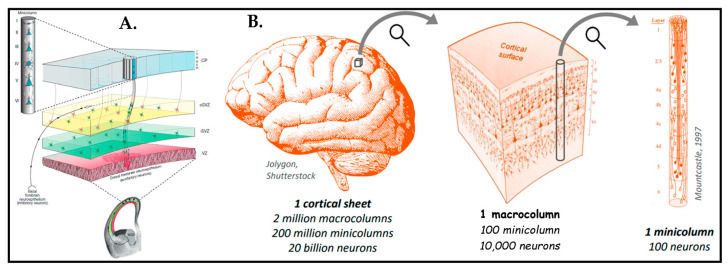
(**A**) Illustration of proliferative zones during neurogenesis and the formation of minicolumns and cortical columns in the prenatal mammalian cerebral cortex. Reprinted with permission from [40] Copyright 2020, Elsevier. (**B**) Schematic of the whole human cortical sheet that has minicolumns (Ø∼50 µm). Each macrocolumn has millions of minicolumns, each one being composed of around 100 neurons. Minicolumns are arranged again in the six layers already depicted in (**A**), with distinct types of neurons and connections per layer. Reprinted from [41] with permission from Matthieu Thiboust.

The human brain has a tremendously organized and unique structure. There are many studies that analyzed the brain cortex by using the Nissl coloring technique that provide proof of neurons revealing a laminar alignment [35,36,37,38]. The cerebral cortex extends into six layers composed of different neuronal cell types orchestrated into particular neural networks during evolution [39]. Figure 1 presents these unique structural features of human brain tissue.

**Figure 2:**
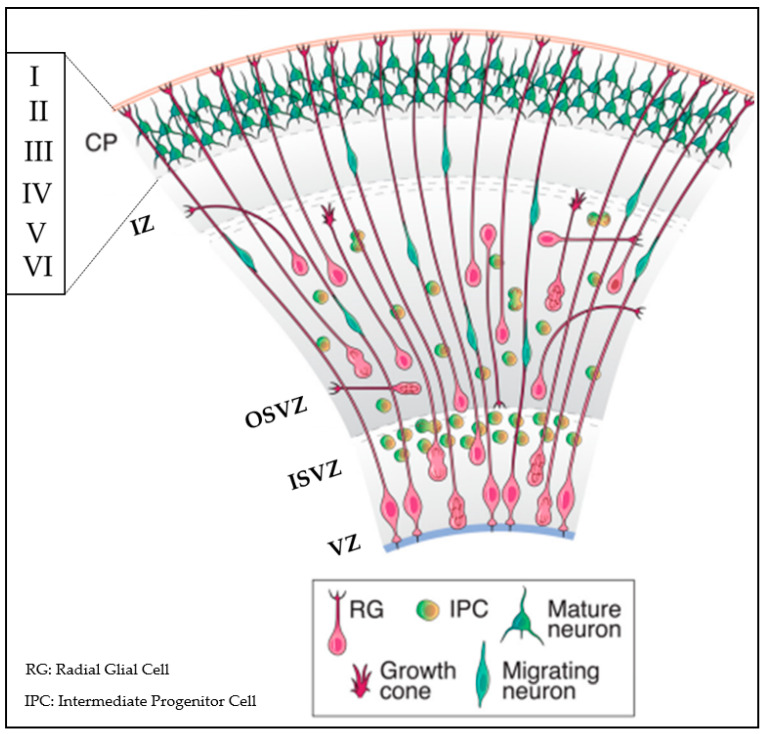
Human cerebral cortex development. The laminated organized cortex that forms six layers (layer I–VI) possesses diverse subtypes of neurons with characteristic functions. Throughout the development of the human cortex, neurons are produced from neural stem cells and progenitors in the ventricular zone (VZ) and the subventricular zone (SVZ). The inner subventricular zone (ISVZ) and outer subventricular zone (OSVZ) is developed by diving into the subventricular zone. While deep layer neurons are forged at early stage, newborn neurons form upper layers such as the intermediate zone (IZ) and the cortical plate (CP). Reproduced with permission from [44] Copyright 2014, Society for Neuroscience.

Figure 2 provides additional insight into the heterogeneity of cell types in the architecture of human brain tissue, which is inclusive of a huge cell variety originated from neuroepithelial cells to radial glial cells, progenitors, immature neurons, differentiated neurons, astrocytes, oligodendrocytes, and microglia [39,42,43]. In this perspective, we take advantage of findings obtained on heterogeneity that could be translated into novel in vitro strategies for mimicking brain mechanics.

### 2.2. Brain Tissue Stiffness

**Figure 3:**
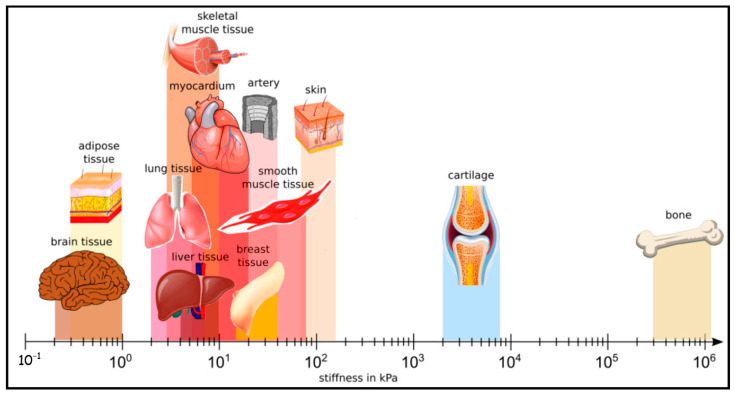
Different human tissues and their stiffness. Brain tissue is one of the softest tissues in the human body. Reprinted with permission from [46] Copyright 2019, Springer Nature.

Next to the unique architecture of the brain, it is noticeably soft. Compared to the other organs in the body, it is one of the softest organs [45]. Figure 3 emphasizes the stiffness difference in various organs in the body [46]. Brain tissue is composed of neuronal cell somata and their protrusions such as dendrites and axons, glial cells, and the extracellular brain matrix (ECM). Until 1971, it was not known that ECM was an asset in brain tissue [47]. Thereafter, the composition and function of the ECM started to become clear fairly recently, with further studies since then [48,49,50]. ECM, as we know it today [51,52], primarily contains glycosaminoglycans (e.g., hyaluronan) that bind water, proteoglycans (e.g., neurocan, brevican, versican and aggrecan), glycoproteins (e.g., tenascin-R), and low levels of fibrous proteins (e.g., collagen, fibronectin). Together with the brain’s high water content (about 80%), there could be a strong correlation between this particular composition of the ECM with the low viscoelastic properties of the brain tissue [53].

**Figure 4:**
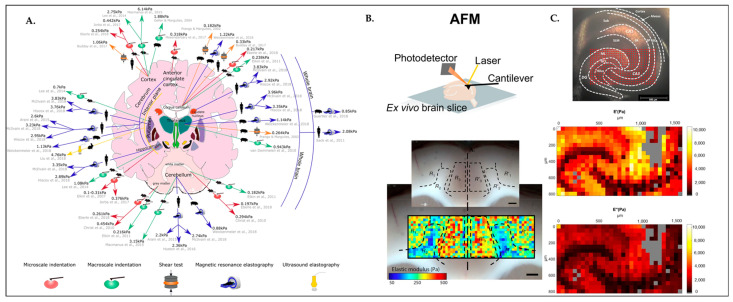
Brain has various regional stiffness. (**A**) Schematic diagram displays stiffness measurements of the healthy adult mammalian brain (human, porcine, rabbit, and rodent, which are illustrated by black silhouette figures) in different regions. Color-coded arrows pointing to the method used to measure brain stiffness in that region. Red, microindentation methods such as atomic force microscopy; green, macroindentation methods; orange, shear tests; blue, magnetic resonance elastography; and yellow, ultrasound elastography. Reprinted with permission from [54] Copyright 2020, European Journal of Neuroscience. (**B**) Stiffness map of the rat cerebral cortex, obtained using atomic force microscopy (AFM). Reprinted with permission from [54] Copyright 2020, European Journal of Neuroscience. (**C**) Figure depicts viscoelasticity maps (E′ = storage modulus; E″ = loss modulus) of the hippocampus of mouse brain slices in Pa over the dentate gyrus (DG) and cornus ammonis (CA3) field. Reprinted with permission from [53] Copyright 2018, Scientific Reports.

Recent works have revealed that brain tissue has high regional stiffness diversity [53,54,55], as can be seen in Figure 4. This correlates with the heterogeneity of the neural and glial cell distribution in layers and their interconnection with the brain extracellular matrix (ECM). Each of these elements has an impact on the local stiffness properties of the brain tissue and hence on how the cells behave in these microenvironments that are considered to be instructive for the cell function. The latter is further detailed in the paragraph below, which offers information from an animal model on the study of the viscoelasticity of the brain tissue microenvironment.

Figure 4A depicts regional stiffness variations in healthy adult mammalian brains performed in vivo or ex vivo by using different methods including atomic force microscopy, magnetic resonance elastography, and ultrasound elastography. In another study, which can be seen in Figure 4B, the AFM micro-indentation technique was performed on a rat brain tissue cortex. Recently, the AFM technique is used to obtain high-resolution stiffness maps in the brain, which gives lots of insights and enlarges our comprehension of regional stiffness changes in the brain, even alterations in response to neurodegenerative diseases, injury, and aging [54]. As represented in Figure 4C, the viscoelasticity map of the hippocampus was obtained from a mouse brain slice performed by oscillatory ramp depth-controlled indentation strokes. It has been reported that the anatomical subregion borders comply well with the morphological heterogeneity of the hippocampus [53].

In conclusion, studies identify brain stiffness as essential for neurobiological processes including neurogenesis [56], axonal pathfinding [57], and the migrational behavior of neurons [58]. Stiffness also correlates with networks’ functional connectivity [59] and synapse formation [60]. Taken together, gaining knowledge of ECM stiffness in brain tissues provides new and essential insights into scaffold design for neural tissue engineering. Moving forward, matrix stiffness is significantly associated with brain injury since the rigidity of the matrix dynamically alters to activate astrocytes for forming a glial scar. Understanding the underlying mechanisms of the ECM stiffness changes, thus becoming a key element to develop in vitro brain injury models [61].

## 3. Neuronal Cell Behavior in Instructive Microenvironments

**Figure 5:**
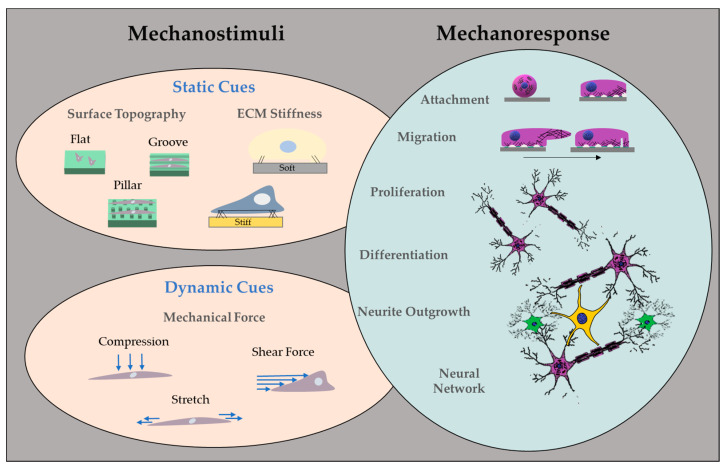
Overview of mechanical cues as instructions in engineered microenvironments.

Several studies have proven that cellular behaviors such as migration, proliferation, and differentiation are highly modulated by their surrounding microenvironment [62,63,64,65]. Cells detect and respond to mechanical stimuli from their environment by changing their phenotypic features such as showing more spread morphology or rounding up due to the substrate stiffness [66], as is also depicted in Figure 5. Multiple studies have investigated how cells react to the stiffness of the material when grown on [67,68].

### 3.1. Impact of Mechanical Forces on Cellular Behavior

The elastic modulus of the native brain is 0.5–1 kPa [69,70] unlike the other tissues such as cartilage (∼500 kPa) [71]. While neurons give in vivo-like responses best to soft materials, they also adhere and survive on stiffer surfaces. As deducted from the stiffness measurements in brain tissues, glial cells such as astrocytes desire slightly stiffer surfaces around 9 kPa, on the contrary to neurons [72,73]. Based on such discoveries in different developmental stages of the brain, neurons extend their axons and dendrites to find their target location. During pathfinding, axons navigate themselves in a well-defined and directed manner over prolonged distances to find their correct targets [57]. Axon guidance has typically been assumed to be regulated by chemical signals [74]. Clearly, physical properties play a role too. Recently, there is growing evidence that mechanical cues are crucial in such processes, including axonal pathfinding, and, subsequently, neuronal migration, differentiation, and, finally, maturation [75].

**Figure 6:**
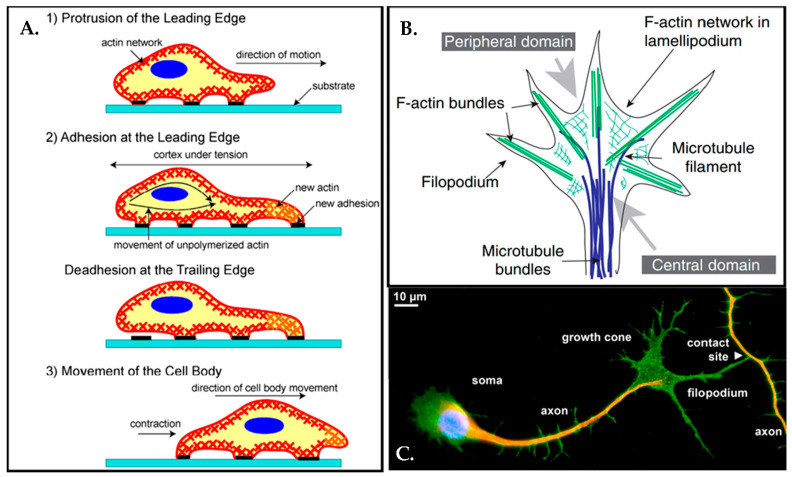
(**A**) A schematic representation demonstrates cell movement on the substrate in three steps. Once the cell extends a projection, the process of cell movement actin starts and actin bundles in the cell membrane begin to reconstruct through the direction of the motion. The cell moves forward by reorganizing the actin network at the leading-edge. Thus, the cell attaches its leading-edge to the substrate followed by detaching the cell body and rear. Traction forces eventually are produced by the actin-myosin network action and the entire cell body moves forward. Reprinted with permission from [76] Copyright 2007, Int. J. Biol. Sci. (**B**) A basic schematic illustration of the growth cone with top view. Reprinted with permission from [82] Copyright 2007, Elsevier. (**C**) Axons can reach incredibly long distances, more than 1.5 m from the spinal cord to the toe for instance. Most of the axons terminate with a few thousand synapses connections and as a result of that perfectly entangled connectivity (1015 synapses). The immunofluorescence micrograph displays a neuron touching another axon with a filopodium at the tip of its growth cone. (green: NCAM1; red: tubulin; blue: nuclear dye). Reprinted from permission from [85] Copyright 2012, Elsevier Ireland Ltd.

From this point of view, axons must apply mechanic force to their environment during expansion [57]. In 1675, Antonie van Leeuwenhoek already monitored cells crawling under the microscope; though, at the time, there was no understanding of the molecular mechanisms behind the motility of the cells. Afterward, it was revealed that the motility of the cell was mainly driven by actin filaments in the cell membrane. Cell movement could be considered in three components: protrusion of the leading edge of the cell, adhesion of the leading edge, adhesion at the cell body and rear, and cytoskeletal contraction to pull the cell forward, as can be seen in Figure 6A [76].

In addition to neuronal movement, neuronal growth can be affected by the mechanics of the environment as shown in vitro [77]. Most recent works demonstrated that neuron growth, neurite extension, and traction forces of the neurons are all regulated and altered by material stiffness [78,79,80,81].

During any such processes, the growth cone of an axon is an extremely active conical shaped structure, which exists in the leading edge of the axons with two different protrusions as filopodia and flat lamellipodia [82] (Figure 6B). Growth cones are crucial for embryonal and adult neurogenesis since they guide axons to their synaptic target to form neuronal connections [83,84] (Figure 6C).

**Figure 7:**
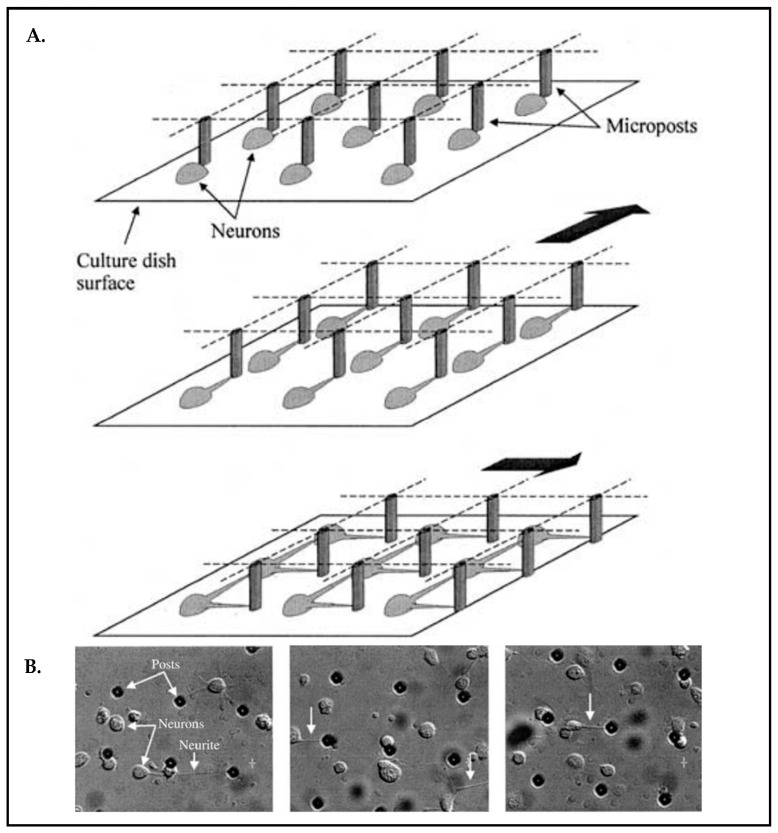
(**A**) Schematic of the neuron connection process with an array of micro-posts. (**B**) Micrographs of embryonic chick neurons seeded. The micro-tool was moved at a constant speed (36 mm/h) to elicit neurites white downward arrows). Reproduced with permission from [89] Copyright 2003, Kluwer Academic Publishers.

Among other methods of stimulation such as electrical and chemical stimuli, mechanical stimuli can exert forces on neuronal cells, influencing their responses. Considering the living organism, cells are subjected to many mechanical restrictions. In fact, all these mechanical processes come into play for the organization of the cell itself, cell–cell, and cell–ECM interactions. It is broadly established that cells adjust themselves to a given mechanical stimulus, but it is questionable whether the resulting behavior is reminiscent of cells and tissues in vivo. To clarify this question, it demands indeed to emulate the mechanical stresses that cells experience in their natural environment in appropriate in vitro model systems. Microsystems technology (alternative terminology: Microelectromechanical systems; short: MEMS) enables novel tools to stimulate cells in vitro. MEMS may apply external forces that might cause influences on cell functions, such as migration, differentiation, maturation, and cell fate [86,87,88,89]. For example, there is a design of a micro-tool reported that creates mechanical pulling on arrayed neuronal cells [89]. The authors’ focus was to achieve a defined and well-controlled connectivity across neuronal networks. The device possesses an array composed of 2500 micro-posts that can be moved laterally (Figure 7A) after axons adhered to the posts and exert the force to arouse neurite outgrowth simultaneously (Figure 7B).

**Figure 8:**
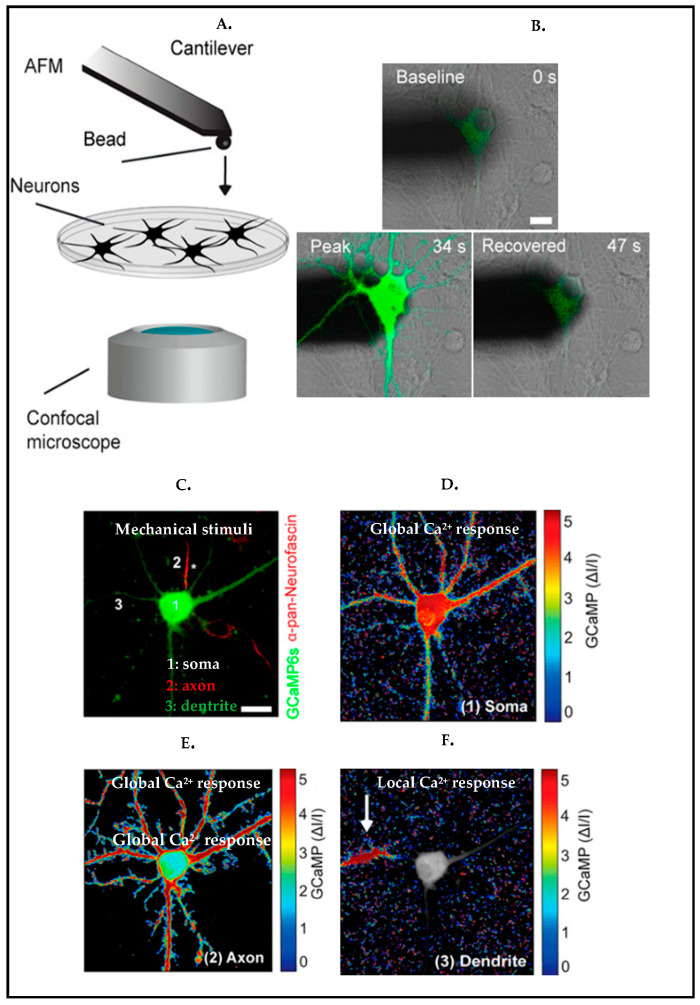
(**A**) Illustration of indention of neuron via AFM and combining with confocal microscopy to monitor the response of the neuron. (**B**) Overlaid differential interference contrast (DIC) to visualize the morphology of the cell, and fluorescence microscopy images to monitor GCaMP6S (green) expressing cortical neurons during stimulation. (**C**–**F**) Normalized fluorescence intensity heat maps display calcium (Ca^2+^) responses to mechanical indentation (**C**) of soma (**D**), axon (**E**), and dendrite (**F**). (**C**) The axon is marked by an asterisk. (green: GCaMP6S, red: anti–pan-neurofascin to stain axon). Scale bars: 10 μm. Reproduced with permission from [90] Copyright 2020, Proceedings of the National Academy of Sciences.

To survey local cell responses upon mechanical poking [90], rat cortical neurons were stimulated via an atomic force microscopy (AFM) indentation technique (Figure 8A). Upon local mechanical stimulation, neurons showed a strong calcium response, and this local stimulus ended up producing a global response all along the neurons, forming a network (Figure 8B). To further investigate how dendrites, somas, and axons unveil their responses differently upon a local stimulus, the study showed that local responses have been propagated forward once a dendrite was stimulated, unlikely in axon and soma, and the response was global along the axon and soma (Figure 8C–F).

### 3.2. Mechanical Guidance in Tissue Model Systems

There is growing evidence showing that mechanical signals play a vital role in various stages of cell development [91,92]. In the literature, numerous methods for creating instructive microenvironments have been detailed, which can be summarized in three main categories: surface topography, synthetic matrix stiffness, and application of external mechanical forces (Figure 5).

**Figure 9:**
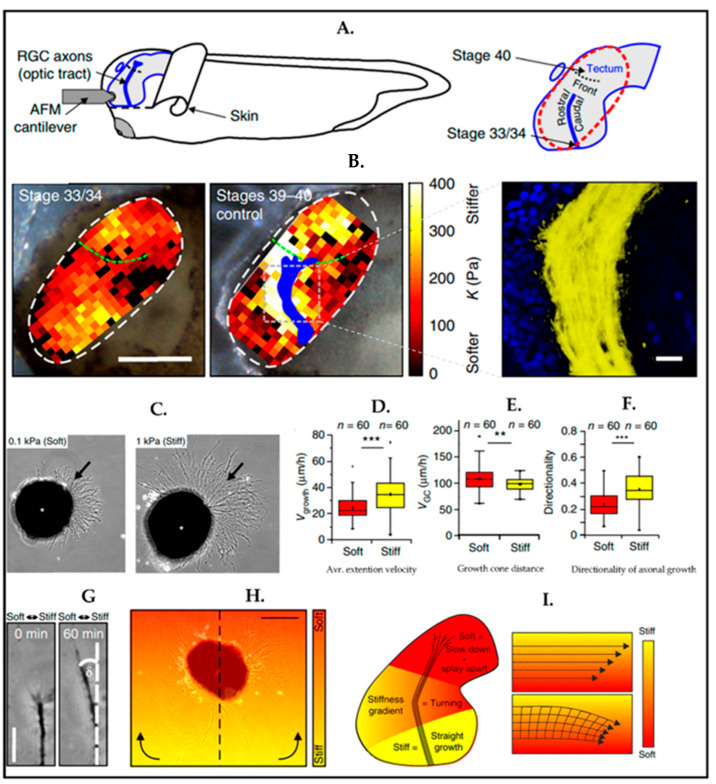
(**A**,**B**) The figure displays in vivo mechanical properties of the Xenopus brain. The area inside the white dashed lines indicates AFM-based stiffness map. Gray lines show immunohistochemistry image of optical track (OT) (cell nuclei (blue), OT (yellow)). (**C**–**F**) Mechanosensitivity of RGC axons in vitro. (**C**) Arrows indicate axons. (**D**) The extension velocity of axons (vgrowth) is higher on stiff substrates (Mann-Whitney test; P = 9.32 × 10^−6^, z = 4.432) (**E**) Growth cones (GC) migrated remarkably faster on soft substrates than the stiff ones. (two-tailed *t*-test; P = 0.00867, t = 2.669) (**F**) Axon growth Is more directed on stiff substrates than on soft substrates. (Mann-Whitney test; P = 1.10 × 10^−6^, z = 4.873). (**C**–**F**) (** *p* = 0.00867; *** *p* < 10^−5^). (**G**–**I**) Time-lapse imaging of axonal growing on a stiffness gradient similarly in vivo data. Eye primordium cells are seeded on a similar stiffness gradient with in vivo. Reproduced with permission from [57] Copyright 2016, Springer Nature.

As indicated in recent works on ex vivo tissue characterization, the mechanical properties of the microenvironment surrounding the cells have an essential role in embryonic development as well as adult brain development. To investigate the impact of tissue stiffness on retinal ganglion cells (RGC) in vivo, Xenopus was exploited as a model. Brain tissue stiffness of the Xenopus was measured by using atomic force microscopy (AFM) at various developmental stages. At each time point, it has been seen that the stiffness of the tissue was heterogeneously distributed, and the direction of the RGC axon growth was perpendicular to the local stiffness gradient of the brain tissue (Figure 9A) [57].

In the same paper, the mechanosensitivity of RGC axons has been studied by using polyacrylamide (PAA) hydrogel in the culture at two stiffnesses: one soft (0.1 kPa) and the other relatively stiff (1 kPa). One day after, the observation was that the axonal growth on the stiff surface was noticeably longer than as on the soft surface (Figure 9B). In addition to the average extension velocity of axons, the absolute distance of the growth cones from their somata and the directionality of the axons have been assessed (Figure 9C–E). Researchers found the axonal growth rate and directionality were higher on stiffer substrates indicative of faster axon movement. On soft substrates, axons explored their environment more thoroughly than on stiff ones. In vivo studies proved that mechanical signals, which have been initiated by a stiffness gradient, were instructive for the cell’s behavior. The experiment has been set up with stiffness gradients, which were similar to data found in vivo. Neurons thus grew to the softer region, as shown in Figure 9G. In the same experiment, the relation between axon turning and stiffness gradient was analyzed (Figure 9H,I) by using time-lapse microscopy, and axons preferred to turn to softer regions.

**Figure 10:**
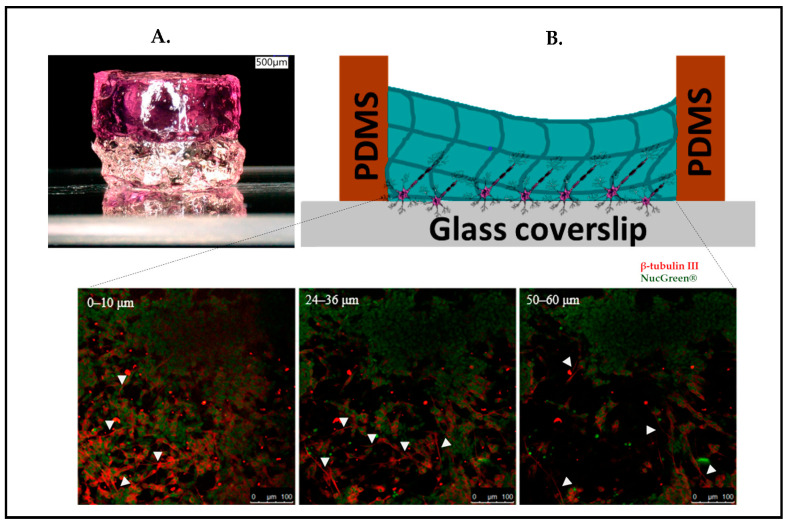
(**A**) Concept of stacked hydrogels cast on a microscope slide. (**B**) Schematic drawing of the SH-SY5Y cells sandwiched in between glass coverslip and hydrogel and confocal microscopy z-stack images from 0 to 60 µm. Neuronal migration into the third dimension. White arrowheads in subfigures (**B**) indicate neuronal outgrowths. (Green: Nucgreen-nuclei; Red: β-tubulin III-axon). Reproduced from [93] Copyright 2021, under the Creative Commons Attribution License.

Our own research on stacked hydrogels [93] also revealed that cells migrate into the softer region even against gravity, which, to some extent, may also simply be triggered by a nutrient gradient of the medium with a higher concentration in the upper regions of the gel. in addition to creating a vertical stiff-to-soft stiffness gradient by molding GelMA atop a glass slide, our concept is aimed to refer to the brain’s layered organizational structure (Figure 10). The study demonstrated that neuronal cells can be sandwiched between the glass and the hydrogel, forming a layered arrangement of three distinct regions: (i) cells remaining in 2D morphology connected laterally on the glass surface, (ii) cells being stretched longitudinally into the z-direction, and (iii) cells fully regaining their rounded morphology such as in vivo with few lateral connections and predominate connections to the lower two layers. In essence, these three regions form a very reductionist model of the microcolumns as depicted in Figure 1B (Section 2). It has been observed that the neuronal cells have the ability to migrate at least 60 µm deep inside the gel layer away from the glass substrates, which are roughly 3–5 times the diameter of the neuronal body in vivo.

**Figure 11:**
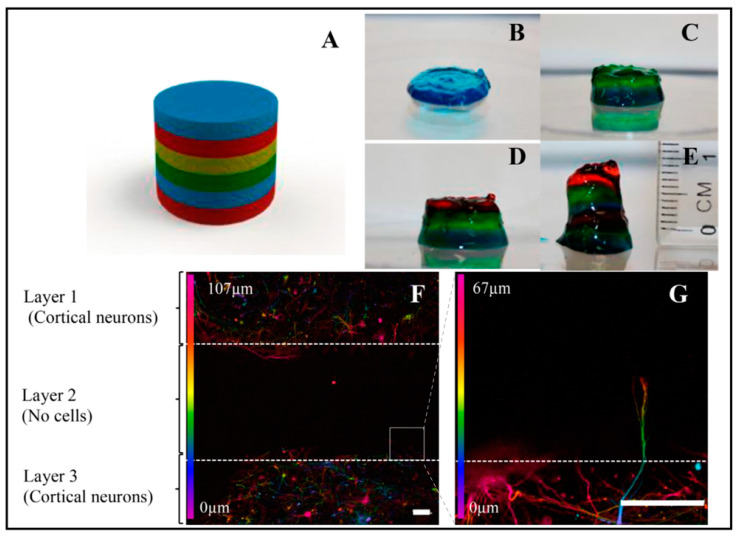
Three-dimensional printed, layered brain-like structures by using peptide-modified gellan gum gel. (**A**) Schematic design by SolidWorks software. (**B**–**E**) The printing process of each layer and each layer was performed with the same gel with different colors. (**F**) Confocal microscopy images, showing axonal extension into another layer on day 5. (**G**) White rectangle displays an enlarged image of (**F**). Reprinted with permission from [94] Copyright 2015, Biomaterials.

Similarly, another exciting model system inspired by the layering of brain tissue was studied by Lozano et al. (Figure 11) [94]. They used a novel peptide-modified biopolymer, the so-called gellan gum-RGD (RGD-GG), by a 3D printing technique, in order to investigate the development of the axons and survival of the cells. Each layer was printed by using 0.5% (*w*/*v*) RGD-GG with different dye colors. As can be seen in Figure 11D, three layers were printed. Cells were encapsulated in layer 1 (top layer) and Layer 3 (bottom layer), while there were no cells in layer 2 (middle layer). The study demonstrated that cortical cell networks arose inside the 3D printed biopolymer comprising cells after 5 days of culture. Furthermore, axons move towards the middle layer (Layer 2, with no cells), as shown in Figure 11F–G. Furthermore, as the method is simple and cost-effective, captured brain-like structures in layers is promising for further applications, including drug testing for the curation of neurodegenerative diseases and cell behavior studies.

**Figure 12:**
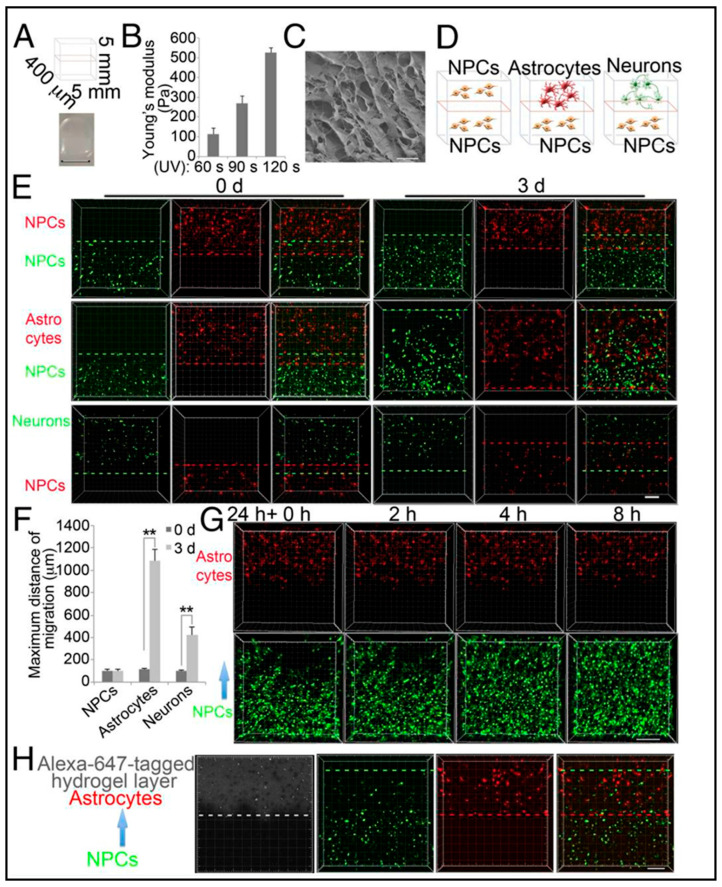
The 3D layered hydrogel system to investigate the migration of hiPSC-derived neural progenitor cells (NPCs). (**A**) Schematic depicts the dimensions of the two-layered hydrogels and the picture at the bottom displays bright-field image of two-layered hydrogels (Scale bar, 5 mm). (**B**) Young’s modulus of HAMA hydrogels measured by atomic force microscope (AFM) following varying UV exposure time. (**C**) Scanning electron microscopy (SEM) imaging of hydrogel (Scale bar, 10 μm). (**D**) Schematic of the two-layered hydrogel shows NPC migration toward NPCs, astrocytes, or neurons. (**E**) Fluorescence microscopy images of NPC migration induced by NPCs, astrocytes, or neurons for indicated times. Between red and green dashed lines depict the farthest migration distance (Scale bar, 200 μm). (**F**) Quantified data of maximum migration distance induced by NPCs, astrocytes, or neurons. Bars represent means; ** *p* < 0.01; *n* = 3. (**G**) Real-time fluorescence microscopy images showing NPC migration toward astrocytes in a time-dependent manner (Scale bar, 200 μm). (**H**) Fluorescence microscopy images of NPC migration induced by astrocytes for 1.5 d with Alexa647-labeled hydrogel to specify the top layer (Scale bar, 200 μm). Reproduced with permission from [96] Copyright 2016, Proceedings of the National Academy of Sciences.

Neuronal migration and maturation are key steps in developing brain tissue since deviations in this process may cause disorders such as autism and schizophrenia [95]. Zhang et al. [96] developed a 3D environment consisting of two-layered hydrogels and human-induced pluripotent stem cell (iPSC)-derived NPCs to reveal MeCP2 dysfunction caused by neuronal migration defects. Their stacks of two hydrogel layers contained methacrylate-modified hyaluronic acid (HAMA), which had a stiffness of about 100 Pa and a pore size of about 10 µm (Figure 12B,C). To inquire more detailed information on NPCs migration in the layers, the NPCs in the top layer were also replaced by either astrocytes or differentiated neurons (Figure 12D). In particular, it was known that astrocytes promote migration [94]. In this in vitro study, hydrogel was chosen to mimic soft brain ECM to accelerate the differentiation of the cells. Using layered hydrogels with cells and co-culturing appeared to be a more realistic model regarding comparison to in vivo cell–cell interactions, which may have actually guided the cellular behavior in the end rather than the gel itself, which essentially acts as a scaffold for cells to adhere to in all three dimensions. In due course of a three-week study described in the same paper by Zhang et al. [96], contrary to the NPCs’ migration into the astrocyte layer (Figure 12G,H), NPCs that were seeded in both layers and NPCs that were seeded in the bottom layer did not migrate (Figure 12E,F). It has been established that these kinds of experimental setups could facilitate neuronal differentiation and maturation by the movement of the cells toward astrocytes (Figure 12G,H).

In addition to stiffness, which is a bulk property of a material, it is also known that the molecular chemical structure forms anchoring points for adherend cells and can act as another important factor in instructive microenvironments such as the extracellular matrix (ECM) of organ tissues, which has been studied already extensively from a tissue engineering perspective, including the fabrication of organoids [97]. Mimicking such cues in modeled ECM systems can be used to inflict a cellular response that helps to culture cells into tissues in a more healthy, in vivo-like manner, thanks to the mechanical cues the cells receive from such structurally modified cell culture substrates. A broad set of substrate preparation and microfabrication methods have been explored to study cells in interaction with topographies. As an initial indication, specifically, neurites have been analyzed in different ways, including counting the number of neurites sprouting from a cell soma, neurites’ average lengths, and their degree of dendritic branching [98,99,100,101,102,103].

**Figure 13:**
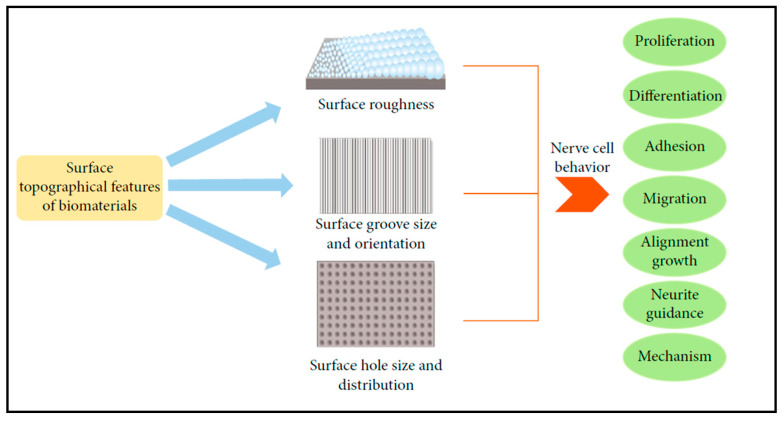
Schematic diagram emphasizes topological features of the materials and their impact on cellular behavior in particular with an emphasis on nerve cell behavior. Reprinted with permission from [106]. Copyright 2021, Fang Liu et al., Stem Cells Int.

Evidence in the literature studying cells in their in vivo ECMs, including neural tissues, shows a diverse range of topographies, from nanometers to micrometers, taking part in the function of the ECM [104,105]. In line with this reasoning, the more the culture surface geometry influences the cell’s response in vitro, the more varied such processes can be exploited to mimic either physiology or pathophysiology in the dish. Figure 13 provides an overview of potential interactive processes triggered by surface topography in a reductionist 2D model system.

**Figure 14:**
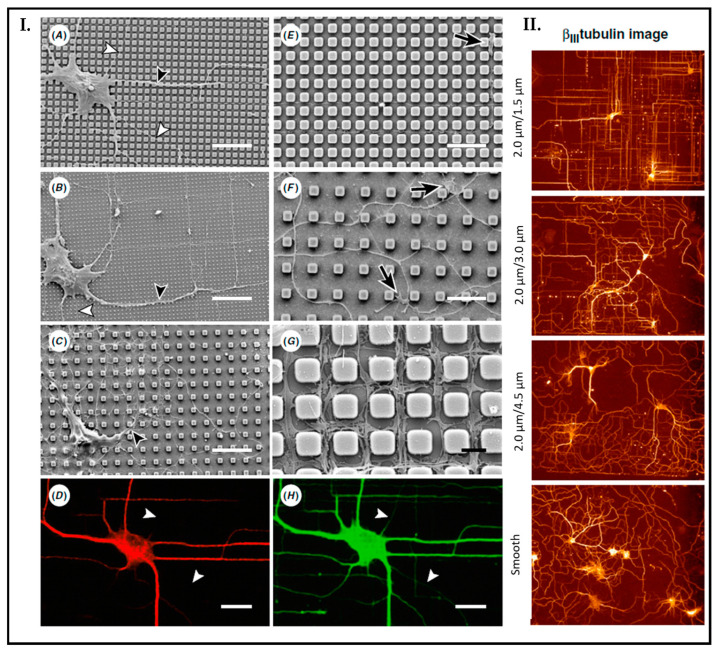
Neuronal growth on a surface with pillar organization. (**A**–**G**) Images have been taken by SEM microscopy, which shows most axons (white arrowhead) and dendrites (black arrowhead). Pillars with pillar width (PW)/pillar gap (PG) (**A**) 2.0 μm/0.5 μm, (**B**) 0.5 μm/1.5 μm, (**C**) 2.0 μm/4.5 μm. (Scale bar = 20 μm). Growth cones (black arrows) on pillars (2.0 μm/1.5 μm) exhibited a narrow profile indicative of rapid growth (**E**). In contrast, growth cones (black arrows) on pillars (2.0 μm/4.5 μm) exhibited a broader profile with a few extending filopodia indicative of slower growth (**F**). Scale bar (**E**,**F**) = 10 μm. (**G**) 2.0 μm/1.5 μm, Scale bar (**G**) = 2 μm. Fluorescent microscopy images with pillar surface (2.0 μm/1.5 μm) by using MAP-2 (**D**) and βIII-tubulin (**H**) to identify axonal growth (white arrowhead) (Scale bar = 20 μm). Fluorescent image of orientation of dendrites and axons on varied pillar width and gap sizes. Red color indicates β_III_-tubulin. The most significant orientation has shown the pillar surface with (PW/PG: 2.0 μm/1.5 μm) (**I**). In Panel II, the fluorescence image depicts the effect of surface topography on the orientation of dendrites and axons. The greatest effect is demonstrated on pillar gaps of 1.5 µm. Random growth is observed on smooth regions. Reprinted with permission from [107] Copyright 2004, J. Neural. Eng.

One such work reported how topographical cues influenced the growth of hippocampal neurons by Dowell-Mesfin et al. [107]. To perform the experiment, they cultured primary hippocampal rat neurons on surfaces with pillars with varied widths and pillar gaps. Their study showed that the neurons’ polarity was significantly affected by topography. Figure 14 (Panel I.) shows the results at the end of 14 days. The cultured samples illustrate if the gap size of the pillar decreases directed orthogonal growth of neurites increases. Contrarily, on smooth surfaces, neurites’ growth orientation is randomly distributed (Figure 14, Panel II.).

**Figure 15:**
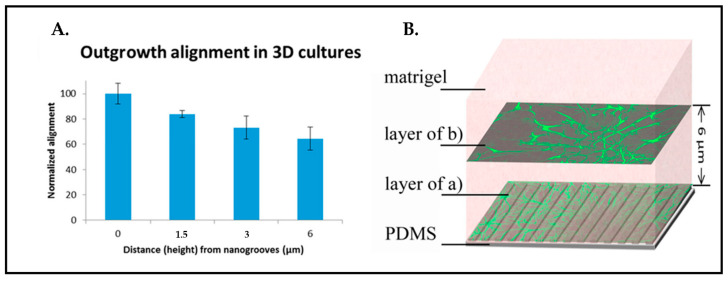
(**A**) Outgrowth alignment is quantified as 65% in 6 µm depth. (**B**) Schematic top view images of immunostained rat cortical astrocytes from 0 µm to 6 µm. (Green: GFAP). Reprinted with permission from [108] Copyright 2015, J. Vac. Sci. Technol.

Three more model systems described previously in the literature are of particular interest related to our own recent research interest in mechanically influencing neural cell responses in three-dimensional (3D) culture systems rather than on surfaces. The investigations performed by our team and described by Frimat et al. [108] illustrated that nanotopographical mechanical cues can influence the alignment of axonal outgrowth deep into the 3D matrix of the Matrigel (Figure 15). Although Matrigel, which was used as a scaffold on top of the nanoimprinted surface topography (here nanogrooves), provides a rich protein and peptide environment, the alignment of outgrowth was shown to be statistically significant when the culture was carried out on the nanogrooves instead of a flat substrate.

Neurons are cell types that must extend to form circuits to survive. Some projections of these circuits run over very long distances to communicate from the central nervous system throughout the body. Patterning of hydrogels is a highly promising technique in terms of neuronal development studies including the observation of neuronal outgrowth, axonal pathfinding, and promoting NPCs migration to its target position. Hydrogel itself enables the creation of a 3D environment for the cells as well as the creation of different topographies. There are many additional properties of hydrogels such as biocompatible, porous structures that allow migration within the environment but also the nutrients and waste exchange and easy fabrication, which make them a preferred matrix material in 3D model system cultures. The most common methods to build structured hydrogels in organ tissue engineering, including the nervous system, are lithography and 3D printing [68,69,70]. In addition, in vitro patterned neurons have been analyzed to initiate neural network circuitry by a controlled topography conceived by microcontact printing [109,110].

**Figure 16:**
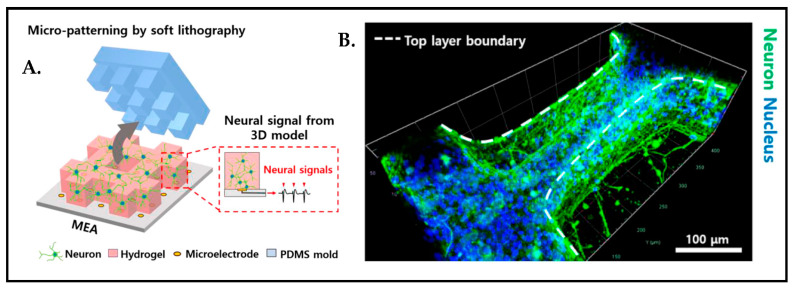
(**A**) Schematic representation of 3D micro-patterned hydrogel model. (**B**) Immunostained image of 8 days cultured neuron distribution in micro-patterned hydrogel. Reproduced with permission from [111] Copyright 2021, IEEE.

A most recent study revealed that the micromolding in capillaries (MIMIC) technique [73] can also be used in order to construct the micro-patterned 3D environment of brain tissue to be able to study the correlation between cell growth orientation and micro-pattern direction (Figure 16).

**Figure 17:**
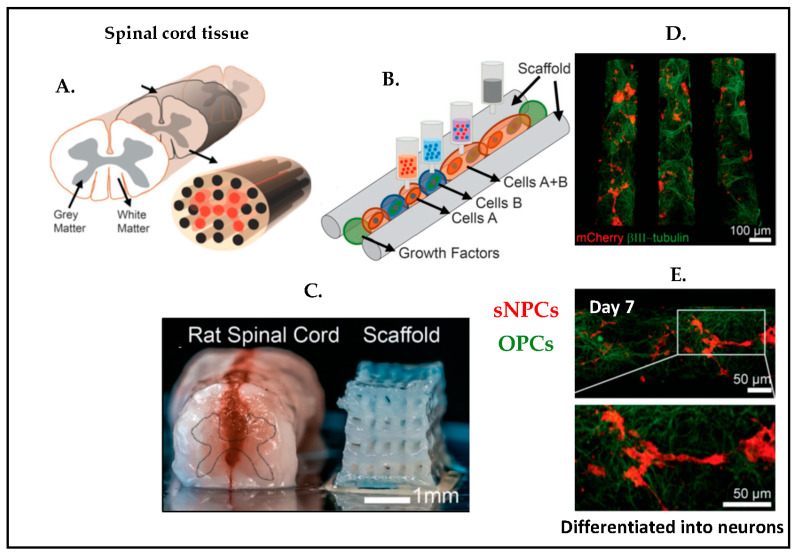
Schematic representation of 3D printing method of the spinal cord tissue. (**A**) Spinal cord illustration. (**B**) Overview of 3D printed tissue with cells. (**C**) Comparison of the rat spinal cord tissue and 3D printed scaffold tissue. (**D**) Immunotstained image of 3D printed scaffold with sNPCs and OPCs on day 4. (**E**) A 7-day culture immunostained image with zoom in. (Green: β3III-tubulin, axonal projections; Red: mCherry, OPCs marker). Reproduced with permission from [112] Copyright 2018, Advanced Functional Materials.

Three-dimensional printing is another promising method for creating an instructive microenvironment raised to study forming and guiding neuronal networks. A 3D printed spinal cord was manufactured [112], which included clusters of induced pluripotent stem cell derived spinal neuronal progenitor cells (sNPCs) and oligodendrocyte progenitor cells (OPCs) (Figure 17). The study demonstrated that sNPCs differentiate and axonal outgrowths trend toward the scaffold channels. This also showed that a combination of OPCs and sNPCs was beneficial to build functional networks.

## 4. State-of-The Art Microfluidic Brain-on-Chip Research

Microfluidic technology becomes important in order to comprehend how mechanical cues might affect neuronal cell dynamics. Early works on cells arranged by a compartmentalized microfluidic layout were presented to generate distinct somata and axonal compartments [113,114]. This work was inspired by even early studies of Robert Campenot, who was the first researcher who introduce two compartments, and, while one was served for culturing neurons, the second one was allowed to observe extending axons [115].

**Figure 18:**
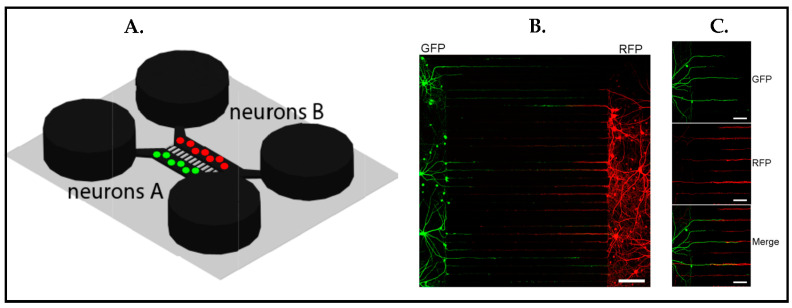
(**A**) Schematic of a microfluidic device that holds two separate neuron populations, microgrooves connecting two compartments. (**B**) Immunostained image displays synapse formations in the microgrooves. Green color expresses green fluorescent protein (GFP)-labeled neurons to visualize dentrites and red color shows red fluorescent protein (RFP)-labeled neurons to illustrate axons. Scale bar 150 μm. (**C**) Zoomed image of (**B**) demonstrating GFP-labeled dendrites penetrating to the microgrooves (top) and RFP-labeled axons (middle) expanding through the microgrooves around 900 μm. The merged image (bottom) shows connection between axons and dentrites. Scale bars = 50 μm. Reproduced with permission from [113] Copyright 2010, Elsevier Inc. All rights reserved.

The original work by Taylor et al. [75] optimized the two-compartment microfluidic device for promoting synapses. Microgrooves between two compartments [114] were utilized to guide dendrites and axons (Figure 18A), and synapse functionalization was verified by immunostaining (Figure 18B) with an enlarged image of Figure 18B (Figure 18C) [113]. One main benefit of the two compartments was enabling independent manipulation in each compartment. For instance, in this study, two distinct neuronal populations such as excitatory and inhibitory could be seeded in two separate compartments in order to monitor synaptic connections across microgrooves. Whereas axons went along the entire microgroove, dendrites grew less than half of the length of the microgrooves into them. Taken together, this compartmentalized microfluidic platform made the easy identification of individual presynaptic and postsynaptic processes within microgrooves possible. This study had significant value in the context of the instructive microenvironment since it demonstrated neuronal organization by guiding axonal and dendritic protrusions in parallel rows via microgrooves between two different cell populations. This idea also became important to mimic different regions in the brain or even bring different organs together in one platform.

These early successes in compartmentalizing neural cultures led scientists to perform novel and sophisticated microtechnology studies, demonstrating a plethora of integrated controllable devices in terms of controlling fluid flow [116], controlling the cell microenvironment (cells embedding in the hydrogel, surface topography, etc.) [117], and controlling concentrations of molecules in space and time since this cannot be captured in traditional Petri dishes [118,119]. The first studies questioning specific neurobiological problems emerged in 2003 [120], and a following study demonstrated that neuronal processes including axonal growth could be directed by fluidically isolated compartments [121]. These studies gave birth to today’s vast range of microfluidic Brain-on-Chip platforms. All these systems aimed to make progress either via rapid prototyping, lowering the cost of fabrication, or enhancing the ease of the interrogation of the cells. Some of these microphysiological system (MPS) products are already commercially available, such as the Organoplate^®^ platform from Mimetas, which relies on bidirectional flow by a rocking plate to generate perfusion. The chip models the blood–brain barrier by using astrocytes and pericytes embedded into a collagen-I hydrogel and endothelial cells to be seeded adjacent to the gel [122]. A further renowned example is the Emulate Spinal Cord Chip that is used to investigate vascular-neural interaction by co-culturing both IPSC-derived brain microvascular endothelial cells (BMECs) and spinal motor neurons [123]. Yet, another commercial product is NeuroFluidics™ from Netri. This chip platform allows working with axonal growth by microchannel between compartments. Next to this, the same product is developed to observe functional activity by integrating with MEA read-out.

Next, we will discuss progress on BoCs with instructive microenvironments found in the literature over the last five years, each representative for a particular class of microfluidic BoC concepts, namely:

1. Microfluidic perfusion to mimic vascular flow

Cerebrospinal fluid (CSF), i.e., the fluid diffusing nervous system tissue, has significant effects on human health. Among others, its main role in the brain is to clear waste products [124,125]. In particular, a pile of unwanted waste proteins in the brain cause such neurological diseases, with the inclusion of Alzheimer’s disease (amyloid beta and tau) and Parkinson’s disease (alpha-synuclein) [126].

**Figure 19:**
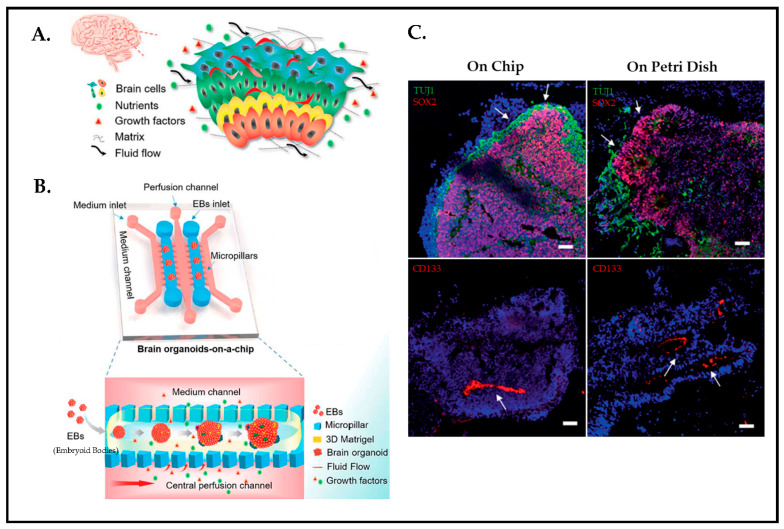
(**A**) Schematic design of brain microenvironment in vivo. (**B**) Chip concept displaying brainoid formation by embedding hiPSCs in Matrigel with fluid perfusion. (**C**) Neurogenesis of brain organoids on chip and Petri dish by immunohistochemical staining on day 33. (Green: TUJ1; Red: SOX2; Red: CD133; Staining is indicated by white arrows. Scale bars: 50 um). Reproduced with permission from [127] Copyright 2018, Royal Society of Chemistry.

To acquire a better understanding of CSF dynamics during early human neurogenesis and its fluid flow driving mechanism, Wang et.al [127] developed a new strategy by utilizing hiPSCs-derived 3D brain organoids and fluid flow together in a microfluidic platform to mimic such a process. The in vivo human brain has inherent control over parameters such as ECM, cellular variety, and fluid flow (Figure 19A,B). At present, although organoids are promising tools to study human brain modeling, organoids are generated by self-organization, which brings batch-to-batch variation and lacks dynamic mechanical cues. Microfluidics, however, offers to model brain tissue in an instructive manner by integrating channels to create fluid flow and controllable microenvironments, including micropillar structures, to steer an organoid formation by facilitating nutrient and oxygen exchange. Figure 19C shows immunostained organoid cultures either under microfluidic or traditional Petri dish conditions. Static cultures “on Petri Dish” and perfused cultures “on Chip” are compared in terms of the effect of the fluid flow on neural differentiation as well as cortical organization. The finding from the immunohistochemical analysis of organoids under controlled fluid flow perfusive conditions improves neuronal differentiation, in vivo organogenesis, and cortical organization compared to static cultures.

2. Integrated perfused vascular tissue to mimic the Blood–brain barrier

The blood–brain barrier (BBB) is a part of the neurovascular unit that mediates communication between the central nervous system and the periphery [128] to maintain brain homeostasis [129]. Furthermore, BBB is a selective physiological barrier [130] acting as the gatekeeper [131] that plays a role in exchanging biological substances, protecting the brain from toxic materials and pathogens, as well as neuronal functions. Deterioration of the BBB is correlated with several neurological disorders in addition to brain injuries. A BBB chip concept is used at Harvard University to recapitulate barrier function and to validate transport drugs and therapeutic antibodies across the human BBB [130]. In addition to this study, scientists at Vanderbilt University developed a novel neurovascular unit that integrated microfluidics and 3D cell culture in order to model human BBB in vitro. The device is composed of a vascular and a brain chamber divided by a porous membrane in the middle. While the brain chamber is critical for growing neurons, astrocytes, and pericytes, and the vascular side allows the seeding of the endothelial cells. Until then, BBB models have failed due to the lack of the flow-generated shear forces to be able to form a tight junction as well as the lack of all the cell types’ involvement [131]. In another study from Vanderbilt University, iPSC-derived human brain microvascular endothelial cells (BMECs) are cultured within 3D gelatin hydrogel with continuous perfusion to model brain microvasculature [132].

**Figure 20:**
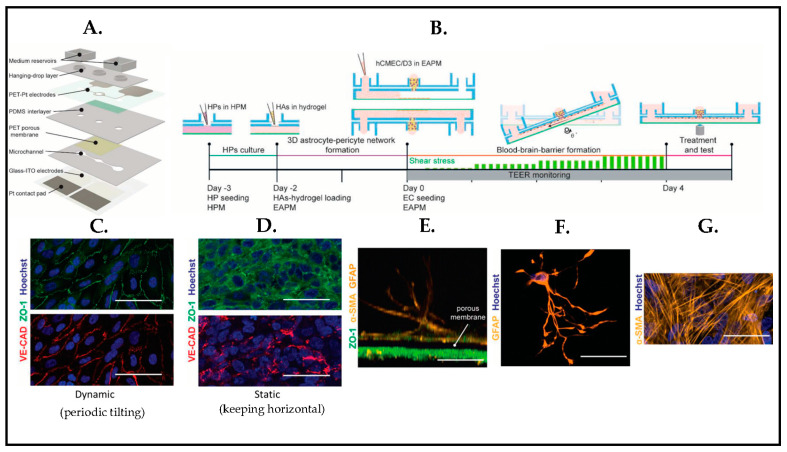
(**A**) Schematic representation of BBB chip. (**B**) Timeline of cell culturing on the BBB chips. (**C**,**D**) Immunostaining images of endothelial cell (EC) monolayer under dynamic and static conditions (Green: ZO-1 (tight-junction protein); Red: VE-CAD (adherent-junction protein); Blue: Hoechst). (**E**,**G**) Human pericyte (HP) cell culture on the opposite side of the membrane in respect of the endothelial cell layer. (Yellow: α-SMA (human pericytes); Blue: Hoechst). (**F**) Astrocyte in a hydrogel, displaying star-shaped morphology in 3D (Yellow: GFAP; Blue: Hoechst). Reproduced with permission from [133] Copyright 2020, Fluids and Barriers of the CNS.

To study barrier response to brain injury, Wei et al. [133] proposed a BBB platform (Figure 20A) that was integrated with transparent electrodes to make real-time monitoring of the barrier. The model consists of human cerebral endothelial cells, primary pericytes, and astrocytes, 3D cultured in a periodically tilting microfluidic platform (Figure 20B). The barrier is eventually characterized by immunostaining under dynamic conditions (Figure 20C), and endothelial cells depicted an elongated morphology and orientation in the flow direction compared to endothelial cells in the static condition (Figure 20D). Confocal microscopy images also confirmed that human pericytes grew on the opposite sides of the porous membrane close to the endothelial cell monolayer (Figure 20E,G), and human astrocytes showed a star shape morphology (Figure 20F). The method offered a highly controlled microenvironment to create an instructive dynamic neurovascular unit (NVU) by integrating different cellular structures comprising microvascular endothelial cells, astrocytes, pericytes, and microglial and neuronal cells with a tilting fluid flow mechanism.

3. Integrated 3D engineered scaffolds enabling organization and mimicry of layers of the brain

**Figure 21:**
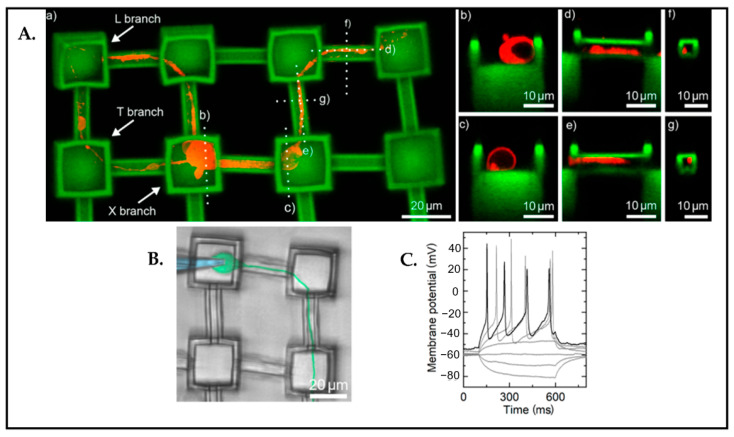
(**A**) Visualization of the neuronal outgrowth in the scaffold by using confocal microscopy z-stacks. (**a**) Red: neurite growing along the microchannels. (**b**–**g**) Dashed lines display cross-sections of the neurite in the channel. (**B**) Demonstration of the patch clamp procedure. (Blue: pipette; Green: cell). (**C**) The measurements of the action potential that are evoked by electrical current injection. Reproduced with permission from [134] Copyright 2020, ACS Nano.

Three-dimensional scaffolding is one of the fundamental approaches in the BoC field in order to imitate the pathophysiology of the brain. Harberts et al. [134] demonstrated 3D nanoprinted microscaffolds for directed neuronal growth and network formations within the scaffold (Figure 21). In the study, they concluded that miniscaffolds contributed to guide neurites, and they showed neuronal functionality with patch clamp measurements.

4. Compartmentalization and integrated organoids

**Figure 22:**
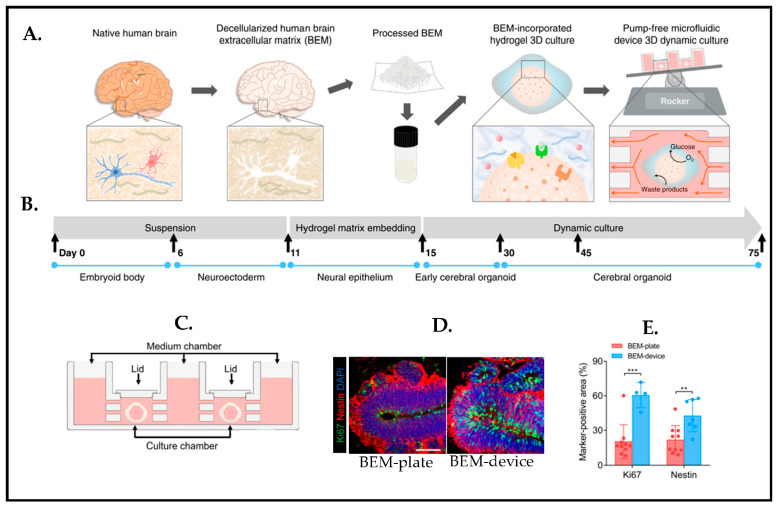
(**A**) Schematic representation of the brain organoid with a combination of 3D decellularized human brain-derived extracellular matrix (BEM) hydrogel culture and the microfluidic device. (**B**) Cerebral organoid fabrication protocol timeline. (**C**) Schematic of the microfluidic device for the brain organoid. (**D**) Immunostaining images for BEM-plate and BEM-device organoids (Green: Ki67 (proliferation marker); Red: Nestin (progenitor marker); Blue: Dapi, scale bars = 50 μm). (**E**) Quantification analyses of markers, Ki67+ and Nestin+ cells in the BEM-plate and BEM-device organoids. Statistical differences between the groups are determined by unpaired two-tailed *t*-test (** *p* < 0.01, *** *p* < 0.001 versus BEM-plate group). Reproduced with permission from [136] Copyright 2021, Nature Communications.

Despite the fact that pluripotent stem cells (PSCs) have the capability to spontaneously self-organize to become organoids, several challenges are still elusive such as a lack of instructive signals during brainoid formation. Spontaneous self-organization also brings batch-to-batch variation, and, therefore, this causes poor reproducibility. There is a need for better functionality, maturation, and obtaining reproducible organoids [135]. To overcome this issue, one study [136] worked on combining engineered human PSC-derived cerebral organoid with a microfluidic device (Figure 22C). The decellularized human brain extracellular matrix (BEM) is utilized by incorporating hydrogel in a microfluidic device that can facilitate nutrient/waste as well as oxygen exchange in order to mimic a cerebrospinal fluid flow in the brain (Figure 22A,B). It eventually stimulates brain-derived extracellular matrix enhanced neurogenesis (Figure 22D), while a microfluidic device with a gravity-driven flow by a laboratory rocker supports the further maturation and recapitulation of cortical organization of brain organoids in a much more precise and reproducible manner (Figure 22E).

Collectively, the overall platform, by having a pumpless and precise fluid exchange, working with small volume, is designed to tackle some restrictions of the current microfluidics containing low throughput 3D cell culture analysis and external pumping systems.

More complex systems require meticulous quality control steps for system validation. Although MPS is a promising candidate to mimic in vivo brain regional architecture and functionality as well as cellular composition, there are several limitations and challenges to address, including standardization and reproducibility [137,138], high throughput, organ–organ interactions, and the integration of vascularization [139] and the immune system. The lack of immune components in the current in vitro models is one of the major limitations as it is key for neuroinflammation in addition to their critical role in the developing brain [138]. In regard to organ–organ interactions, or in another word, the combination of different organs into one model system known as either human-on-chip or body-on-chip approach [140,141] has become important in terms of chemical toxicity such as liver metabolic activity or filtering function of the kidney.

Furthermore, a planar multi-electrode array (MEA) is a standard tool that is used to assess neuronal functionality by the electrical activity of neurons. However, this tool has an issue with 3D models. For instance, organoids touch only a few of the electrodes and also only signals of the most outer layer of the organoids can be recorded without slicing them. Hence, it is hard to get reproducible results from plate to plate. Solutions might be either to use MEAs with needle-like electrodes, i.e., 3DMEA, by which the organoid tissues can be penetrated into or to grow the organoids directly around a mesh of (nano)electrodes, which might help to get recordings not only from the surface but also from the inside, as discussed by Passaro et al. [142].

## 5. Conclusions and Perspectives

In this review, recent advances in brain research have been underlined for developing an instructive microenvironment of brain modeling in vitro. Based on the knowledge that neuronal cells can sense and give a response to mechanical cues both internally and externally in their microenvironment, we categorized the mechanical cues by topography, a stiffness gradient, and externally applied mechanical cues such as directly exerting a mechanical force by pocking or pulling.

Even though principles differ regarding how neuronal cells are being influenced by these mechanical signals, it has been known that neuronal cell behavior consisting of proliferation, migration, axonal outgrowth, differentiation, and neural network formation is crucially affected by such cues. Mechanical stimulation hence is a promising toolbox for brain research.

Notwithstanding huge advances in vitro human brain model, there are several challenges remaining before their implementation in clinical translation can be warranted, including the creation of a relevant brain model, building complexity and scaling, and functional verification. However, the better the brain is understood the more closely the real physiological environment can be established. Integration of high-throughput technologies to measure certain brain functions in real-time and under vastly changing drug screening conditions, including the implementation of different types of genetic modified tissues for mechanism of action studies comparing healthy and disease state functions, can benefit from advances in micro-biofabrication methods. Considering refinements in the BoC field from using animal-derived cells to hiPSCs, as well as the intervention of the industry in the field, the development of new platforms has accelerated.

Finally, we can summarize that the careful engineering of the microenvironment matters in providing a ground truth to human brain modeling in a dish. Microfluidic BoCs are clearly providing an asset to neurobiological research in vitro by helping to standardize and automate the culture conditions.

## Data Availability

Data sharing is not applicable to this article.
